# Supplementary material for: Differentially evolved glucosyltransferases determine natural variation of rice flavone accumulation and UV-tolerance
Source: Nat Commun. 2017 Dec 7;8:1975. doi: 10.1038/s41467-017-02168-x (PMC5719032; doi:10.1038/s41467-017-02168-x)
Supplement: Supplementary file 1 — Supplementary Information [file 41467_2017_2168_MOESM1_ESM.pdf]

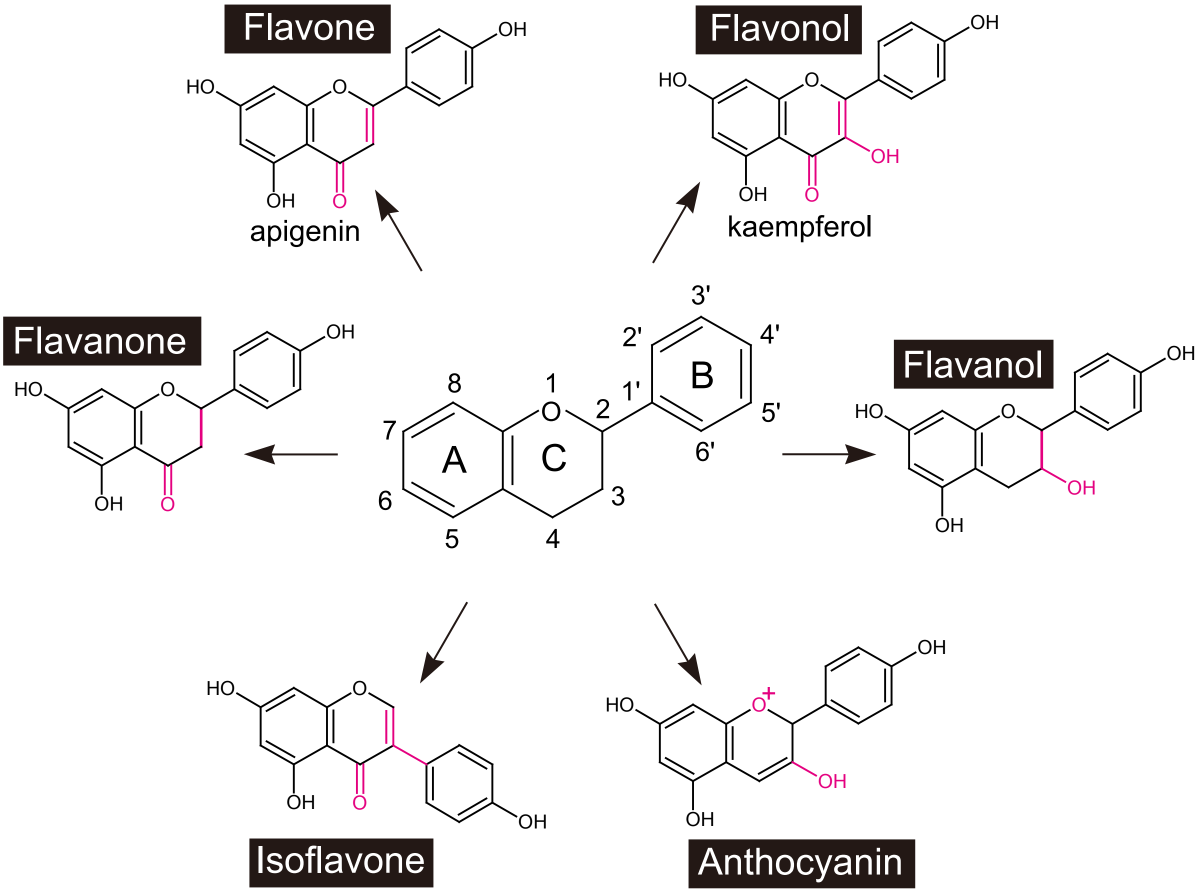

Supplementary Fig. 1 | Nuclear structure and classification of flavonoids. The skeleton structure consists of A, B and C rings with group positions numbered. flavonoids are sub-divided according to the hydroxylation pattern and conjugation between the aromatic rings, as indicated in magenta. The structures of six simple flavonoids are showed. Apigenin and kaempferol are two substrates widely used in this study.

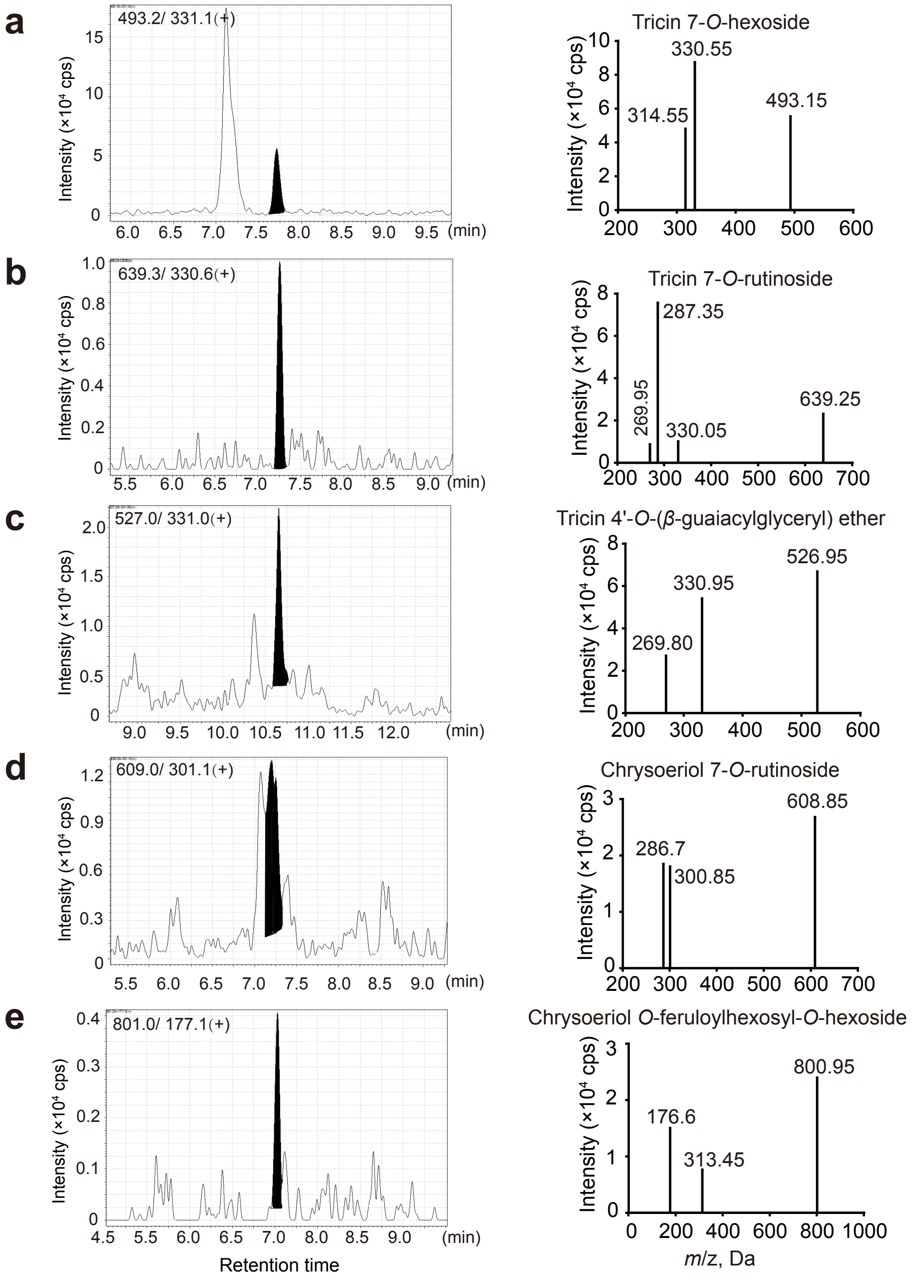

Supplementary Fig. 2 | Detection of flavones by target LC-MS method in Arabidopsis leaf. Extracted ion chromatogram (left) and MS/MS spectra (right, main fragments) of *m/z* 493.2 (**a**), 639.3 (**b**), 527.0 (**c**), 609.0 (**d**) and 801.0 (**e**) by positive mode.

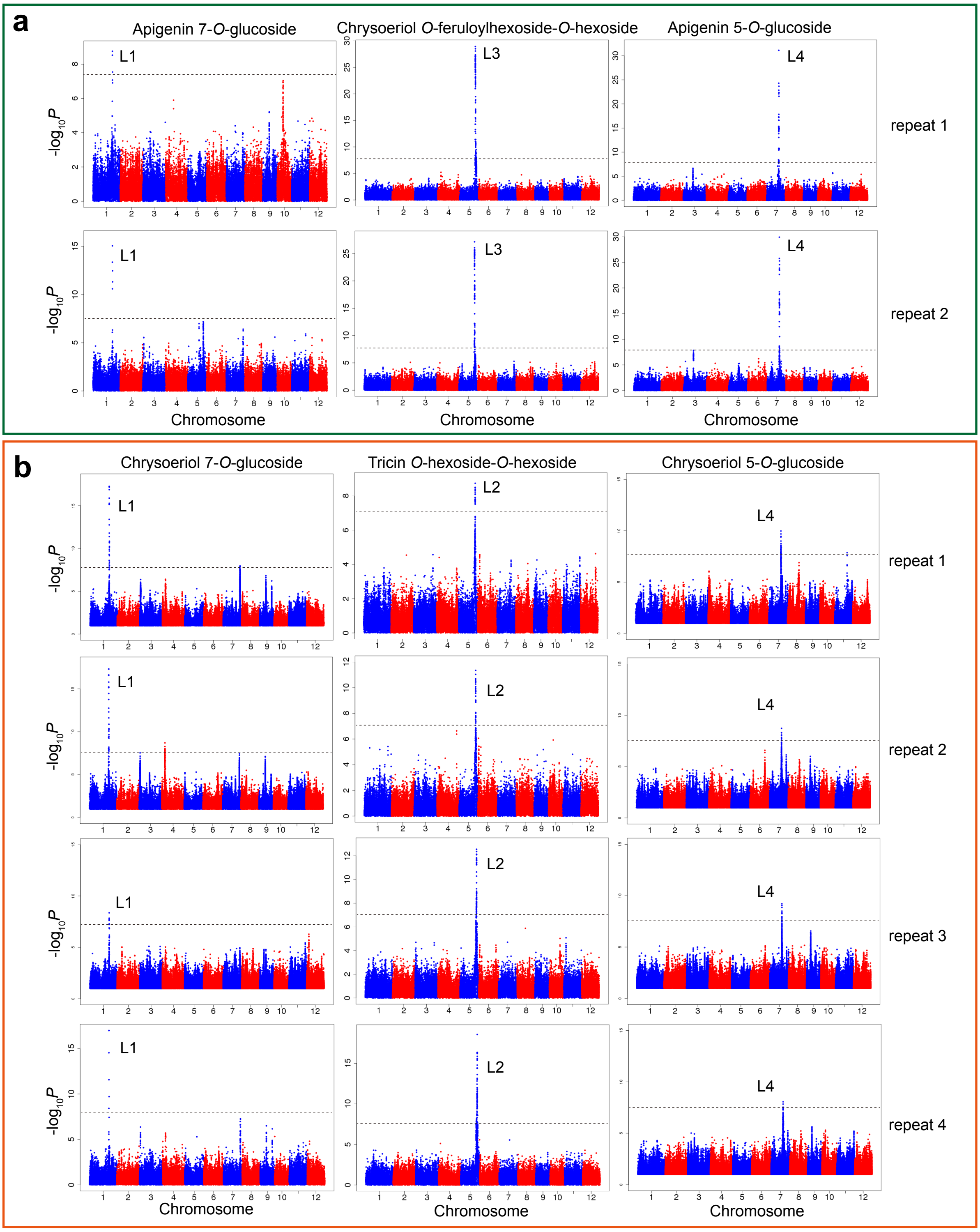

Supplementary Fig. 3 | Manhattan plots of 6 typical flavonoids detected in rice. The strength of association is indicated as the negative logarithm of the *P* value for the linear mixed model. All metabolite-SNP associations with *P* values below 6.6×10^{-8} (horizontal dashed lines in all Manhattan plots) are plotted against genome location in intervals of 1 Mb. Green box indicates the leaf tissue (**a**) and orange box indicates the seed tissue (**b**).

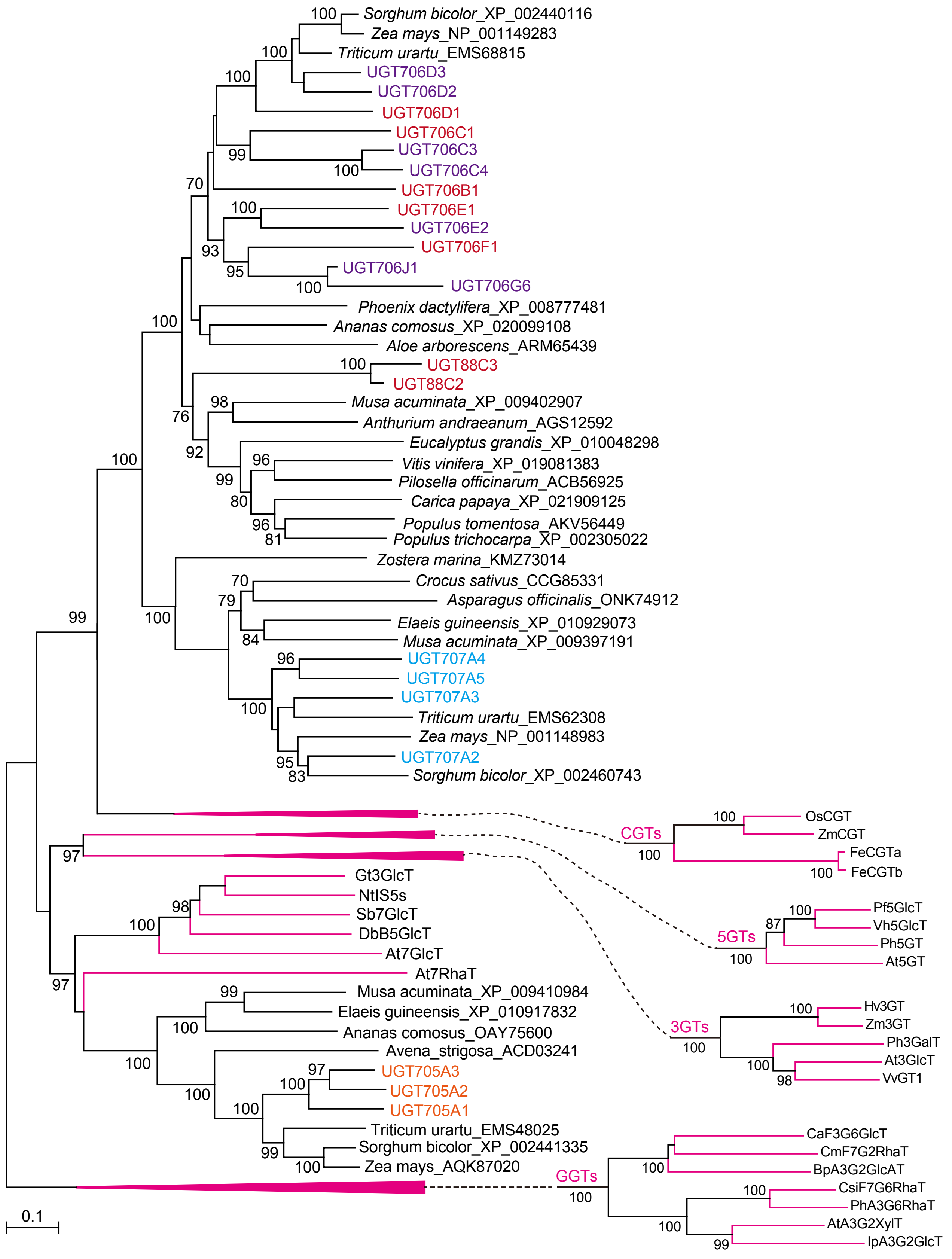

Supplementary Fig. 4 | Phylogenetetic analysis of flavonoid UGTs. An unrooted phylogenetic tree was constructed as described in Methods. Bootstrap values >70% (based on 1,000 replications) are indicated at each node (bar: 0.1 amino acid substitutions per site). Reported flavonoid UGTs are indicated in magenta branches. Candidate UGTs from L1, L2, L3 and L4 are indicated in red, orange, purple and blue, respectively.

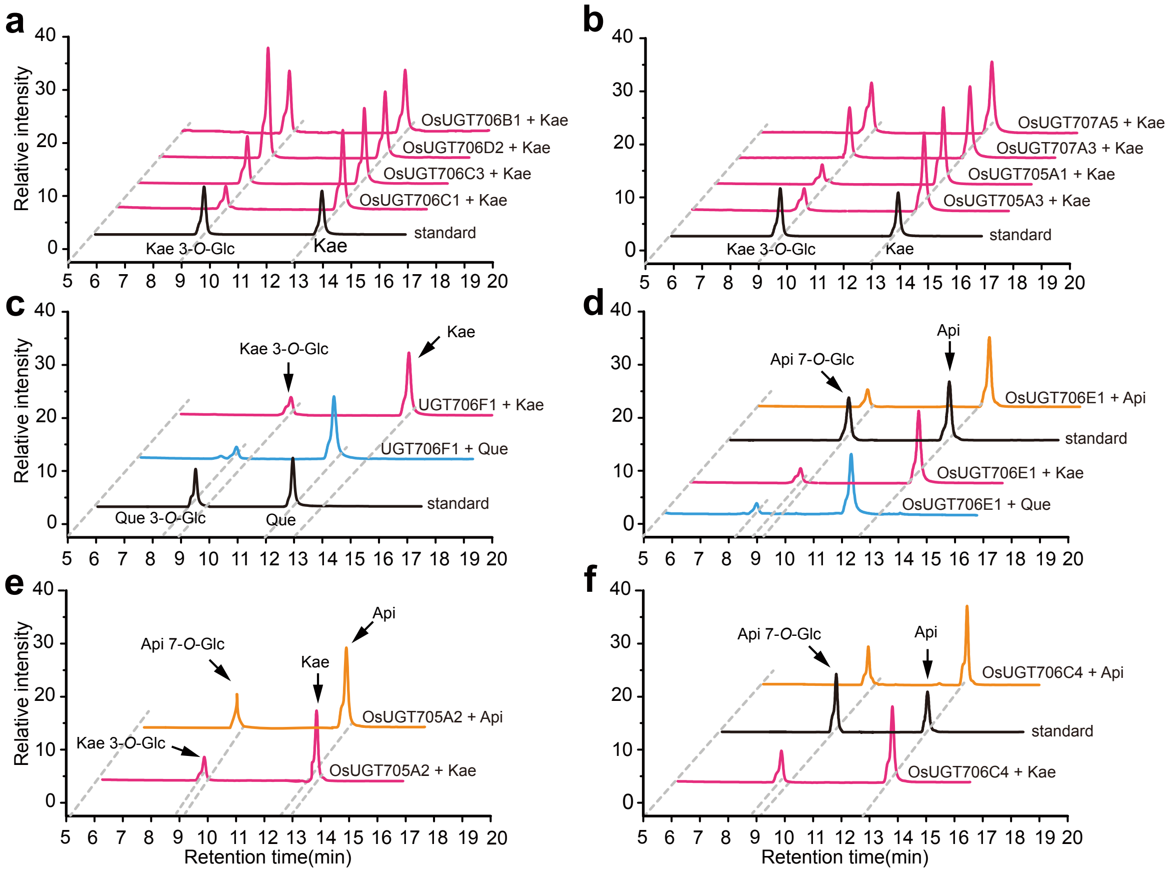

Supplementary Fig. 5 | Enzymatic assays of recombinant flavonoid UGT proteins. HPLC chromatograms of reaction of flavonol UGTs with kaempferol and quercetin (**a** - **c**) and flavone UGTs with apigenin and kaempferol (**d** - **f**). Kae, kaempferol, indicated in magenta. Que, quercetin, indicated in blue. Api, apigenin, indicated in orange. Glc, glucoside.

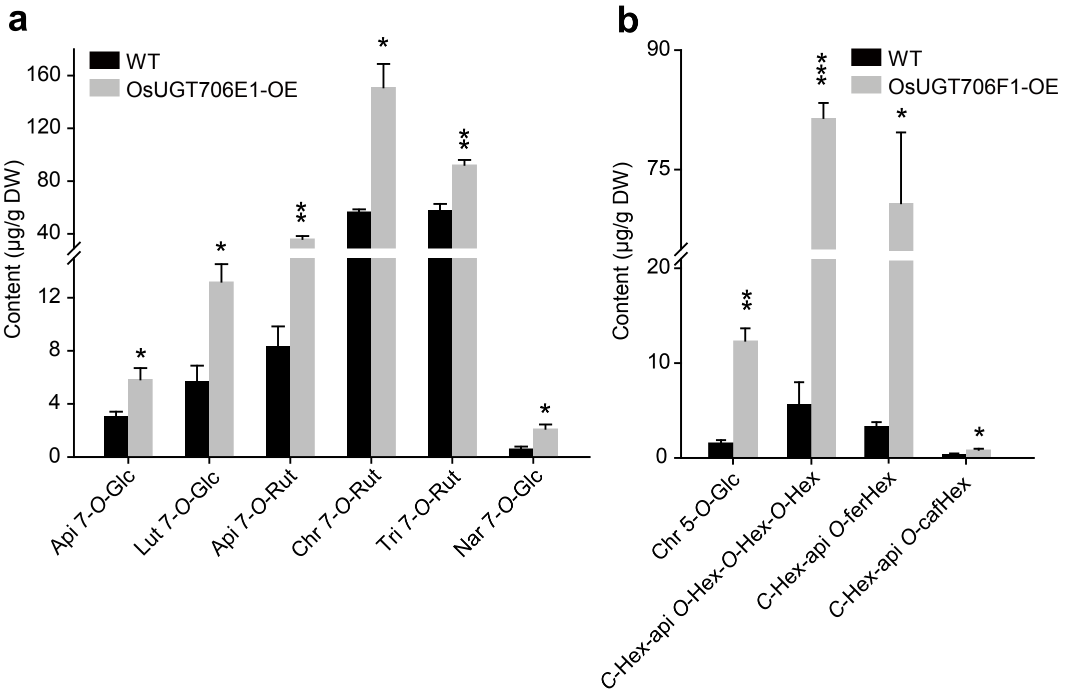

Supplementary Fig. 6 | Metabolite analyses of transgenic individuals in rice. Bar plots for the amounts of corresponding flavonoid glycosides in OsUGT706E1 (**a**) and OsUGT706F1 (**b**) overexpression (OE) plants. WT, the transgenic background variety ZH11. Data are presented as the mean ± SD, n = 3. *P < 0.05, **P < 0.01, ***P < 0.001, Student's t-tests. Api, apigenin; Lut, luteoin; Chr, chrysoeriol; Tri, tricin; Nar, naringenin; Glc, glucoside; Rut, rutinoside; Hex, hexoside.

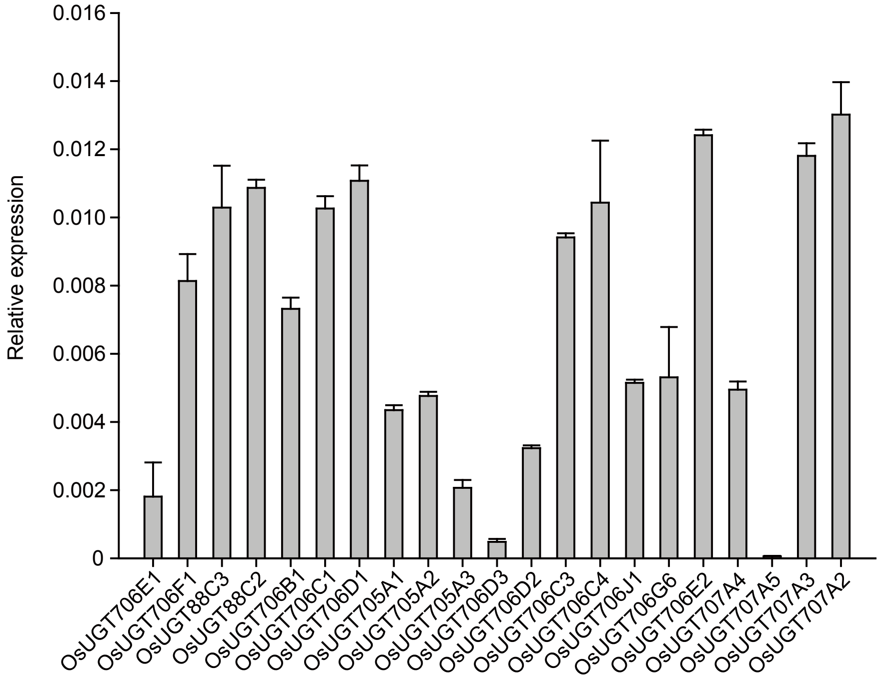

Supplementary Fig. 7 | Transcription abundance of flavonoid UGTs in rice flag leaf. Bar plots for the relative expression of flavonoid UGTs in rice leaf. *OsUbq13* was chosen as internal standard for normalization. Data are presented as the mean ± SD, *n* = 3.

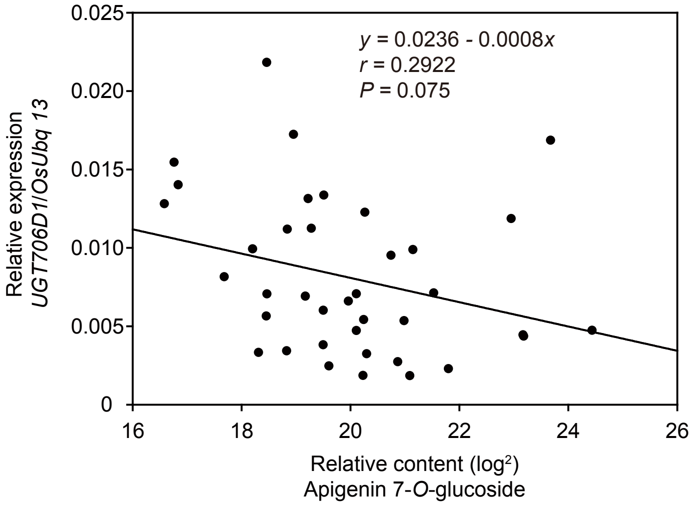

Supplementary Fig. 8 | The correlation between *OsUGT706D1* transcriptional level and the content of apigenin 7-*O*-glucosides in 39 rice varieties. The *r* value is based on the Pearson correlation coefficient. The *P* value is calculated using the Student's *t*-tests.

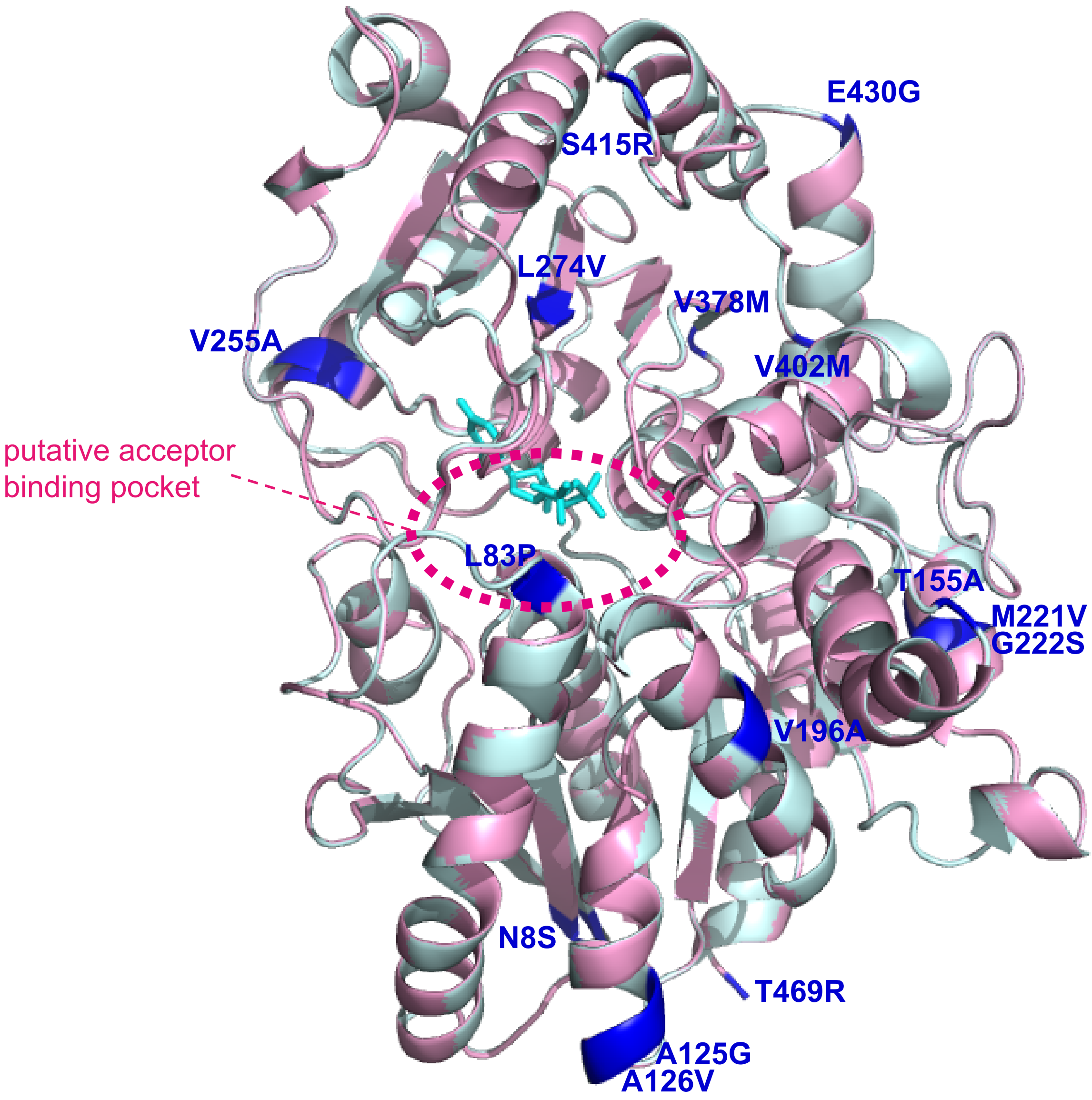

Supplementary Fig. 9 | Distribution of 15 amino acids in the model structure of UGT706D1. UGT706D1 structure was modeled by online service (https://swissmodel.expasy.org/). Two allelic protein were shown and aligned in pink and light cyan, respectively. UDP molecue (in cyan) and the putative acceptor binding pocket (in magenta) were marked on the base of reported structure (2acv), UGT71G1. 15 amino acid substitutions are labled with their positions (in blue).

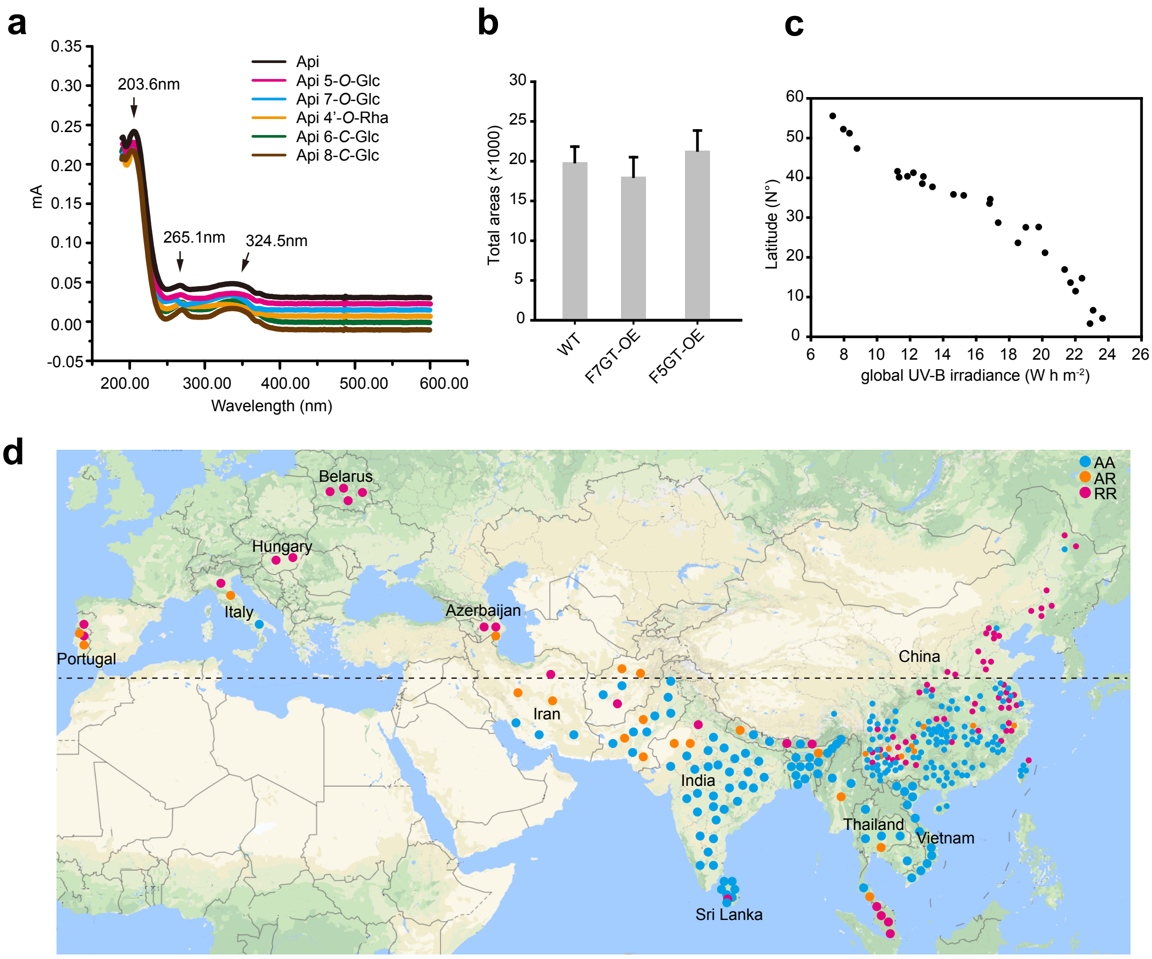

Supplementary Fig. 10 | Functional characterization of the role of flavone glucosides. (**a**) UV absorbance profile of apigenin and apigenin glycosides measured by HPLC-PDA. similar λ_{max} were indicated by arrows (203.6 nm, 265.1 nm and 324.5 nm). Api, apigenin. Glc, glucoside. Rha, rhamnoside. (**b**) Total flavonoids measured by HPLC at 330 nm in transgenic plants and WT. Data are presented as the mean ± SD, n = 3. (**c**) Plot for relationship between global UV-B irradiance and latitude in Europe and Asia. (**d**) Geographical distribution of the two-gene combinations in 345 rice varieties in European and Asia. The different colors represent different combinations of *OsUGT707A2* and *OsUGT707A2*. R is the reference allele, and A is the alternate allele. Map is modified from Google Maps.

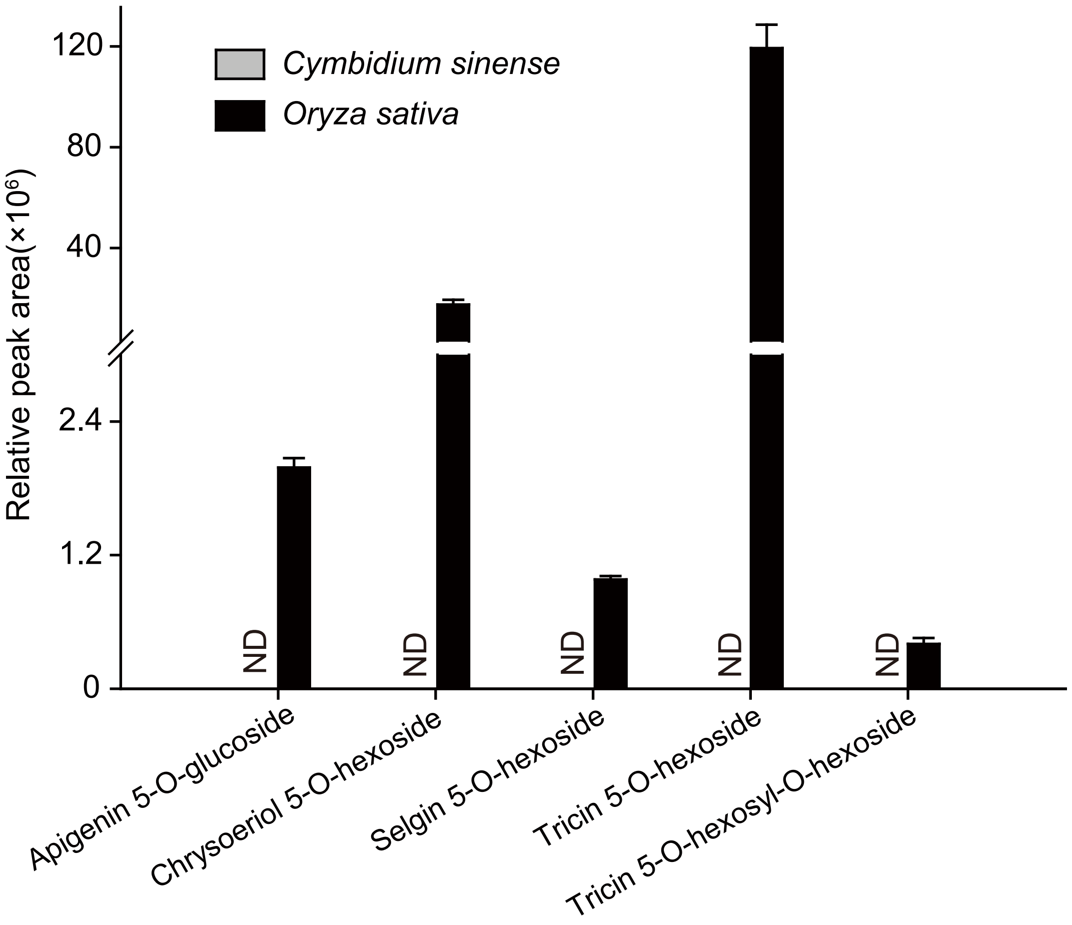

Supplementary Fig. 11 | Profiling of flavone 5-*O*-glucosides in *Cymbidium sinense* and *Oryza sativa*. LC-MS profiling of flavone 5-*O*-glucosides in the leaf of *Cymbidium sinense* and *Oryza sativa*. Data are presented as the mean ± SD, *n* = 3. ND, not detected.

Supplementary Table 1. Kinetic parameters of flavonol UGTs.

Substrate[a]	k_{cat} (s^{-1})	K_m (μM)	k_{cat}/K_m
**OsUGT706B1**			
Kaempferol	0.08 ± 0.01	7.7 ± 0.5	0.01
Quercetin	ND	ND	ND
**OsUGT706C1**			
Kaempferol	0.1 ± 0.01	20.7 ± 2.5	0.005
Quercetin	ND	ND	ND
**OsUGT706C3**			
Kaempferol	0.3 ± 0.06	33.4 ± 2.6	0.01
Quercetin	0.1 ± 0.05	34.8 ± 4.2	0.003
**OsUGT706D2**			
Kaempferol	0.2 ± 0.01	6.7 ± 1.4	0.03
Quercetin	0.5 ± 0.04	60.5 ± 8.0	0.008
**OsUGT706F1**			
Kaempferol	0.2 ± 0.06	24.7 ± 2.0	0.008
Quercetin	0.05 ± 0.004	10.5 ± 3.5	0.005
**OsUGT707A3**			
Kaempferol	0.1± 0.03	11.1 ± 1.8	0.01
Quercetin	ND	ND	ND
**OsUGT707A5**			
Kaempferol	0.1 ±0.05	32.7 ± 3.8	0.003
Quercetin	ND	ND	ND

[a]1.5mM UDP-glucoside was used as sugar donor.

All the data are presented as mean ± SD from two replicate independent assays.

ND, not determined due to the low activity.

Supplementary Table 2. Kinetic parameters of flavone UGTs.

Substrate[a]	k_{cat} (s^{-1})	K_m (μM)	k_{cat}/K_m
**OsUGT705A2**			
Apigenin	0.07 ± 0.01	7.9 ± 1.2	0.01
Luteolin	ND	ND	ND
Kaempferol	0.08 ± 0.01	3.8 ± 0.3	0.02
Quercetin	ND	ND	ND
**OsUGT706C4**			
Apigenin	0.2 ± 0.1	33.6 ± 3.2	0.006
Luteolin	ND	ND	ND
Kaempferol	0.3 ± 0.04	15.8 ± 0.8	0.02
Quercetin	0.1 ± 0.03	20.7 ± 3.7	0.005
**OsUGT706D1**			
Apigenin	0.7 ± 0.02	17.7 ± 1.3	0.04
Luteolin	0.4 ± 0.2	11.6 ± 0.35	0.04
Kaempferol	0.4 ± 0.01	16.8 ± 1.3	0.02
Quercetin	0.07	11.9 ± 1.9	0.001
**OsUGT706E1**			
Apigenin	0.5 ± 0.2	7.2 ± 1.2	0.06
Luteolin	ND	ND	ND
Kaempferol	0.09 ± 0.01	1.9 ± 0.2	0.05
Quercetin	0.1 ± 0.02	14.6 ± 2.2	0.007
**OsUGT707A2**			
Apigenin	0.4 ± 0.06	8.9 ± 1.8	0.05
Luteolin	0.05 ± 0.01	1.5 ± 0.5	0.03
Kaempferol	0.20 ± 0.01	6.7 ± 0.7	0.03
Quercetin	0.06 ± 0.01	5.1 ± 0.6	0.01

[a]1.5mM UDP-glucoside was used as sugar donor.

All the data are presented as mean ± SD from two replicate independent assays.

ND, not determined due to the low activity.

Supplementary Table 3. Kinetics of OsUGT707A2 from two alleles.

	OsUGT707A2-allele I[a]			OsUGT707A2-allele II[b]		
	k_{cat} (s^{-1})	K_m (μM)	k_{cat}/ K_m	k_{cat} (s^{-1})	K_m (μM)	k_{cat}/ K_m
Api[c]	0.44 ± 0.059	8.87 ± 1.79	0.050	0.29 ± 0.089	32.08 ± 2.27	0.0090

[a]allele I was amplified from high content of apigenin 5-*O*-glucoside variety.

[b]allele II was amplified from low content of apigenin 5-*O*-glucoside variety.

[c]UDP-glucose is accepted as the sugar donor.

All the data are presented as mean ± SD from two replicate independent assays.
